# The Activities and Secretion of Cytokines Caused by Delamanid on Macrophages Infected by Multidrug-Resistant *Mycobacterium tuberculosis* Strains

**DOI:** 10.3389/fimmu.2021.796677

**Published:** 2021-12-24

**Authors:** Xia-Li Lyu, Ting-Ting Lin, Jing-Tao Gao, Hong-Yan Jia, Chuan-Zhi Zhu, Zi-Hui Li, Jing Dong, Qi Sun, Wei Shu, Li-Ping Pan, Zong-De Zhang, Qi Li

**Affiliations:** Beijing Chest Hospital, Capital Medical University, Beijing Tuberculosis and Thoracic Tumor Research Institute, Beijing, China

**Keywords:** multidrug-resistant *Mycobacterium tuberculosis* (MDR-MTB) strains, macrophages, cytokines, Delamanid (Dlm), immune mechanism

## Abstract

**Background:**

Delamanid (Dlm) is an effective drug against drug-susceptible and drug-resistant *Mycobacterium tuberculosis* strains, including Multidrug-resistant *Mycobacterium tuberculosis* (MDR-MTB). There are few reports on the activity and secretion of cytokines caused by Dlm on macrophages infected by MDR-MTB strains. Therefore, this article aims to observe the bactericidal activity and secretion of cytokines of the macrophages infected by MDR-MTB strains after Dlm was administered, so as to provide a basis for further perfecting the mechanism of Dlm.

**Methods:**

Samples were respectively collected to count the intracellular colony-forming unit (CFU) of macrophages infected by MDR-MTB or H37Rv strains at 4, 8, 24, and 48 h after Dlm at MIC, 10MIC, and 20MIC were administered. Samples were respectively collected to detect the level of IL-12/23 p40, TNF-α, IL-6, and IL-10 in the culture supernatant of macrophages infected by MDR-MTB or H37Rv strains at 4, 24, and 48 h after Dlm at MIC were administered. The levels of four cytokines in the culture supernatant were measured using the Luminex^®^ 200™ (Luminex, USA) according to the manufacturer’s instructions. Data were analyzed by SPSS 25.0 software. The continuous data in normal distribution were expressed as mean ± standard deviation ( $\bar{x}$ ± *s*) and analyzed by *t* or *F* test. *P*<0.05 was considered statistically significant.

**Results:**

(1) After Dlm was applied to macrophages infected by MDR-MTB strains:

(A) The intracellular CFU gradually decreased, reached the lowest value at 48 h, and was lower than that of Dlm before administration and infection group (*P*<0.05). (B) The intracellular CFU was further reduced after increasing Dlm dose to 10MIC and 20MIC, and the latter was lower than that of the former (*P*<0.05). (C) The intracellular CFU of MDR-MTB group was higher than that of H37Rv group at 4~48 h after administration (*P*<0.05). (2) After Dlm at MIC dose was applied to macrophages infected by MDR-MTB strains: (A) The level of IL-12/23 p40 at any time didn’t change compared with that of Dlm before administration (*P*>0.05), while the level of IL-12/23 p40 at 4 h was higher than that of the infection group (*P*<0.05). The levels of TNF-α at 24 and 48 h were higher than that of Dlm before administration (*P*<0.05), but were similar to that of the infection group (*P*>0.05). In addition, the levels of IL-12/23 p40 and TNF-α at any time were similar to that of the H37Rv group after administration (*P*>0.05). (B) The levels of IL-6 at 24 and 48 h were higher than that of Dlm before administration (*P*<0.05), but were similar to that of H37Rv group (*P*>0.05) and were lower than that of infection group (*P*<0.05). The level of IL-10 at any time didn’t change compared with that of Dlm before administration (*P*>0.05), but was lower than that of the infection group at 4~48 h and was lower than that of the H37Rv group at 24 h (*P*<0.05). (C) The level of IL-12/23 p40 and IL-10 didn’t change with the change of intracellular CFU (*P*<0.05), while the level of TNF-α and IL-6 increased with the intracellular CFU decreasing, and the increase level of TNF-α was lower than that of the infection group (*P*<0.05).

**Conclusions:**

Dlm had strong bactericidal activity against intracellular MDR-MTB, which was time-dependent and concentration-dependent. Its bactericidal activity against intracellular MDR-MTB strains was weaker than that against drug-susceptible tuberculosis strains. Dlm might have immunomodulatory effect, inducing low expression of Th2 cytokines IL-6 and IL-10 at different times after administration.

## Introduction

One of the most formidable challenges in modern public health is the emergence and pervasiveness of drug-resistant diseases. Among them, multidrug-resistant tuberculosis (MDR-TB) is caused by *Mycobacterium tuberculosis* (MTB) resistant to both isoniazid and rifampin (1). The drug resistance types of multidrug-resistant *Mycobacterium tuberculosis* (MDR-MTB) include phenotype resistance and gene resistance. Based on sputum acid-fast staining smear, sputum *MTB* culture, identification of bacteria type, drug sensitivity test, and Xpert MTB/RIF^®^ assay, only 55% of tuberculosis patients can be etiology confirmed according to 2019 World Health Organization (WHO) report, while only 37% can be etiology confirmed in China (1). Among them, Kat G and rpoB gene of isoniazid and rifampin mutation genes usually caused high drug resistance of MDR-MTB (2, 3). The treatment of MDR-TB and rifampicin-resistant tuberculosis (RR-TB) is more difficult. The global treatment success rate for MDR/RR-TB patients is about 57%, and the treatment success rate in China is only 52% according to the report of the World Health Organization (WHO) in 2020 (4). Therefore, the development of new anti-tuberculosis drugs is an important strategy for tuberculosis control, especially for drug-resistant tuberculosis. In drug development, some biological methods to simulate the interaction between the host and pathogens will help to understand the drug-resistant mechanism of MDR-MTB strains, the efficacy and mechanism of new anti-tuberculosis drugs, so as to provide better treatment options of MDR-TB control (1).

Delamanid (Dlm) mainly inhibits cell wall synthesis by inhibiting phthioic acid biosynthesis (5), which is effective against drug-susceptible and drug-resistant *Mycobacterium tuberculosis* (including MDR-MTB) strains (6). The clinical trials of Dlm-containing chemotherapy regimens have entered in the phase III according to the report by WHO in 2020 (4), and the clinical efficacy of Dlm and Dlm-containing chemotherapy regimens have been reported (6, 7). However, there are few reports on the effect of Dlm on macrophages infected by MDR-MTB strains and its mechanism (1). Therefore, this article aims to observe the bactericidal activity of Dlm on macrophages infected by MDR-MTB strains. Based on some cytokines participating in the activation, phagocytosis, sterilization, and other functions of macrophages, we chosen four Th1/Th2 cytokines including Interleukin-12/23 p40 (IL-12/23 p40), Tumor Necrosis Factor-α (TNF-α), Interleukin-6 (IL-6), and Interleukin-10 (IL-10) to observe the expression change after Dlm acting on macrophages infected by MDR-MTB strains, so as to provide a basis for further perfecting the mechanism of Dlm.

## Materials and Methods

### Compounds, THP-1 Cells, and Bacteria

Delamanid (Dlm, S5007) was purchased from Selleck. Alamar Blue was purchased from BD. Magnetic Luminex^®^ Assay was purchased from R&D. Dlm was dissolved in DMSO at final stock concentration. The preparation solutions were stored at −80°C without freeze-thaw cycles. THP-1, H37Rv (ATCC_27294), and MDR-MTB strains (Clinical number: 24635; PNB test: *Mycobacterium tuberculosis*; drug sensitivity test by proportional method: isoniazid and rifampicin were drug-resistant, while other drugs were drug-sensitive; full gene sequencing results: only rpoB gene and katG gene mutation) were obtained from Beijing Chest Hospital, Capital Medical University.

### THP-1 Cell Culture, Differentiation, and Bacterial Infection

THP-1 cells were maintained in RPMI-1640 supplemented with 10%FBS at 37°C in a humidified incubator of 5% CO_2_. They were differentiated into macrophages by incubation with 100 ng/ml PMA for 36~48 h, and then were ready for experiments (8, 9). Cell adherence (about 70~80%) was observed under optical microscope. MTB was inoculated into 7H9 medium (containing 10% OADC and 0.05% Tween-80) and cultured at 37°C to the logarithmic growth phase (OD_600_≈0.6–1.0). An ultrasonic disperser was used to disperse and count MTB. Differentiated macrophages were infected with MTB at a multiplicity of infection (MOI) of 10:1 for 4 h. MOI (10:1) was selected for references (8, 9). *In vitro* infection experiments using MTB were conducted in BSL2 level facilities in the Department of Bacteriology (ITEM: LA2-6A1 Class II BSC per NSF 49, ULPA filter, ISOCIDE), Beijing Tuberculosis and Thoracic Tumor Research Institute after approval by the Institutional Biosafety Committee of China (Asset number: 100408).

### Broth Microdilution Minimal Inhibitory Concentration Method

The microplate Alamar Blue assay (MABA) method was used to determine the MIC of Dlm for H37Rv strains. Briefly, the turbidity of the resulting MTB suspension was adjusted with sterile deionized water to an equivalent of McFarland standard 1 suspension (~5×10^7^ CFU/ml) and then diluted 1:50 (~1×10^6^ CFU/ml) in 7H9 broth medium. The 2× inoculum was poured into a disposable inoculum reservoir, and then 100 µl was transferred to the microtiter plate wells using an eight-channel micropipette and sterile tips with filters. The final inoculum size in each was ~5×10^5^ CFU/ml. The growth control well, but not the well containing the negative control, was also inoculated with MTB. Dlm was diluted and tested by the Clinical and Laboratory Standards Institute (CLSI) guidance. Then 32.5 μl of staining agent (20 μl Alamar Blue and 12.5 μl of 20% Tween-80) was added to each well after incubating for 7 days at 37°C. Microtiter plates were read by visual inspection at 24 h after inoculation to establish MIC for Dlm. MIC was defined as the lowest concentration of the drug that prevented the color from changing from blue to pink. The MIC of Dlm was 0.05 mg/L.

### Measurement of MTB Infection by CFU Assays

Briefly, differentiated macrophages were infected with MTB at a multiplicity of infection of 10 for 4 h and washed with PBS for four times. Then, the final concentrations of Dlm at 1X, 10X, and 20X MIC value were added to each well acting for 4, 8, 24, and 48 h, washed and lysed with PBS containing 0.5% Triton X-100. CFU assays were performed by harvesting serially diluted suspensions on Middlebrook 7H10 agar plates supplemented with 10% OADC at 37°C for 3~4 weeks to quantify the number of MTB that had infected the macrophages.

### Magnetic Luminex^®^ Assays

Briefly, differentiated macrophages were infected with MTB at a multiplicity of infection of 10 for 4 h and washed with PBS for four times. Then, the final concentrations of MIC were added to each well acting for 4, 24, and 48 h, then the cell-culture supernatants were collected. The levels of IL-12/23 p40, TNF-α, IL-6, and IL-10 in cell-culture supernatant were measured using the Luminex^®^ 200™ (Luminex, USA) according to the manufacturer’s instructions.

### Statistical Analysis

The data were analyzed by SPSS 25.0 software and Prism 8.0 software. The continuous data were expressed as mean ± standard deviation ( $\bar{x}$ ± *s*) and analyzed by *t* or *F* test. *P*<0.05 was considered statistically significant.

## Results

### Intracellular CFU in the Macrophages Infected by MDR-MTB Strains After Dlm Was Administered for 4~48 h

Dlm exhibited the potent anti-tuberculosis activity against *M. tuberculosis* H37Rv and MDR-MTB strains in macrophages. The characteristic of bactericidal activity was assessed by determining the CFU reduction after Dlm was administered with different doses for 4~48 h. After Dlm was applied to macrophages infected by H37Rv or MDR-MTB strains, the results of intracellular CFU count were shown in **Table 1**. As shown in **Table 1**, the intracellular CFU gradually decreased in the macrophages infected by MDR-MTB or H37Rv after Dlm at MIC was administered for 4~48 h, which were lower than that of Dlm before administration and infection group (no-drug group) at 48 h except for the intracellular CFU of MDR-MTB group at 4 h after administration, and reached the lowest value at 48 h (*P*<0.05). Increasing Dlm dose to 10MIC and 20MIC could further reduce the intracellular CFU in the macrophages infected by H37Rv or MDR-MTB strains (*P*<0.05) at 48 h. In addition, the intracellular CFU of H37Rv group with dose of 20MIC was similar to that with dose of 10MIC (*P*>0.05) at any time, while the intracellular CFU of MDR-MTB group with dose of 20MIC was lower than that with dose of 10MIC (*P*<0.05) at any time.

**Table 1:** Intracellular CFU in the macrophages infected by MDR-MTB after Dlm was administered ( $\bar{x}$ ± *s*).

	4 h before administration	4 h after administration	8 h after administration	24 h after administration	48 h after administration	*F* test	*P* value
H37Rv strains							
Control	120.00 ± 4.00	104.67 ± 9.87	114.00 ± 5.29	102.67 ± 13.61^#^	85.33 ± 6.43^#&♣*^	7.111	0.006
MIC	120.00 ± 4.00	54.67 ± 12.86^a#^	36.00 ± 4.00^a#&^	30.67 ± 10.07^a#&^	24.00 ± 4.00^a#&^	72.941	0.001
10MIC	120.00 ± 4.00	52.00 ± 10.58^a#^	25.33 ± 9.24^a#&^	18.67 ± 2.31^a#&^	10.67 ± 2.31^ab#&♣^	132.798	0.001
20MIC	120.00 ± 4.00	38.67 ± 2.31^a#^	26.00 ± 6.00^a#&^	11.33 ± 1.16^ab#&♣^	9.33 ± 1.16^ab#&♣^	522.144	0.001
* F* test	–	26.502	132.484	72.114	242.306	–	–
* P* value	–	0.001	0.001	0.001	0.001	–	–
MDR-MTB strains							
Control	114.67 ± 7.57	92.67 ± 6.43^#^	102.67 ± 4.62	96.67 ± 11.55^#^	83.33 ± 5.77^#♣^	7.128	0.006
MIC	114.67 ± 7.57	92.00 ± 8.00^#^	84.67 ± 4.16^a#^	69.33 ± 9.45^a#&♣^	50.00 ± 5.29^a#&♣*^	34.549	0.001
10MIC	114.67 ± 7.57	84.67 ± 4.16^#^	74.00 ± 12.17^a#^	64.67 ± 4.16^a#&^	30.00 ± 9.17^ab#&♣*^	43.864	0.001
20MIC	114.67 ± 7.57	52.00 ± 10.58^abc#^	44.67 ± 9.87^abc#^	30.00 ± 4.00^abc#&^	16.00 ± 6.93^abc#&♣^	65.317	0.001
* F* test	–	18.917	24.944	35.102	52.972	–	–
* P* value	–	0.001	0.001	0.001	0.001	–	–

Control: No drug on macrophages infected by MTB. ^#^The P value of 4 h before administration and other group was less than 0.05. ^&^The P value of 4 h after administration and other group was less than 0.05. ^♣^The P value of 8 h after administration and other group was less than 0.05. *The P value of 24 h after administration and other group was less than 0.05. a: The P value of control and other group was less than 0.05. b: The P value of MIC and other group was less than 0.05. c: The P value of 10MIC and other group was less than 0.05.

**Figure 1:**
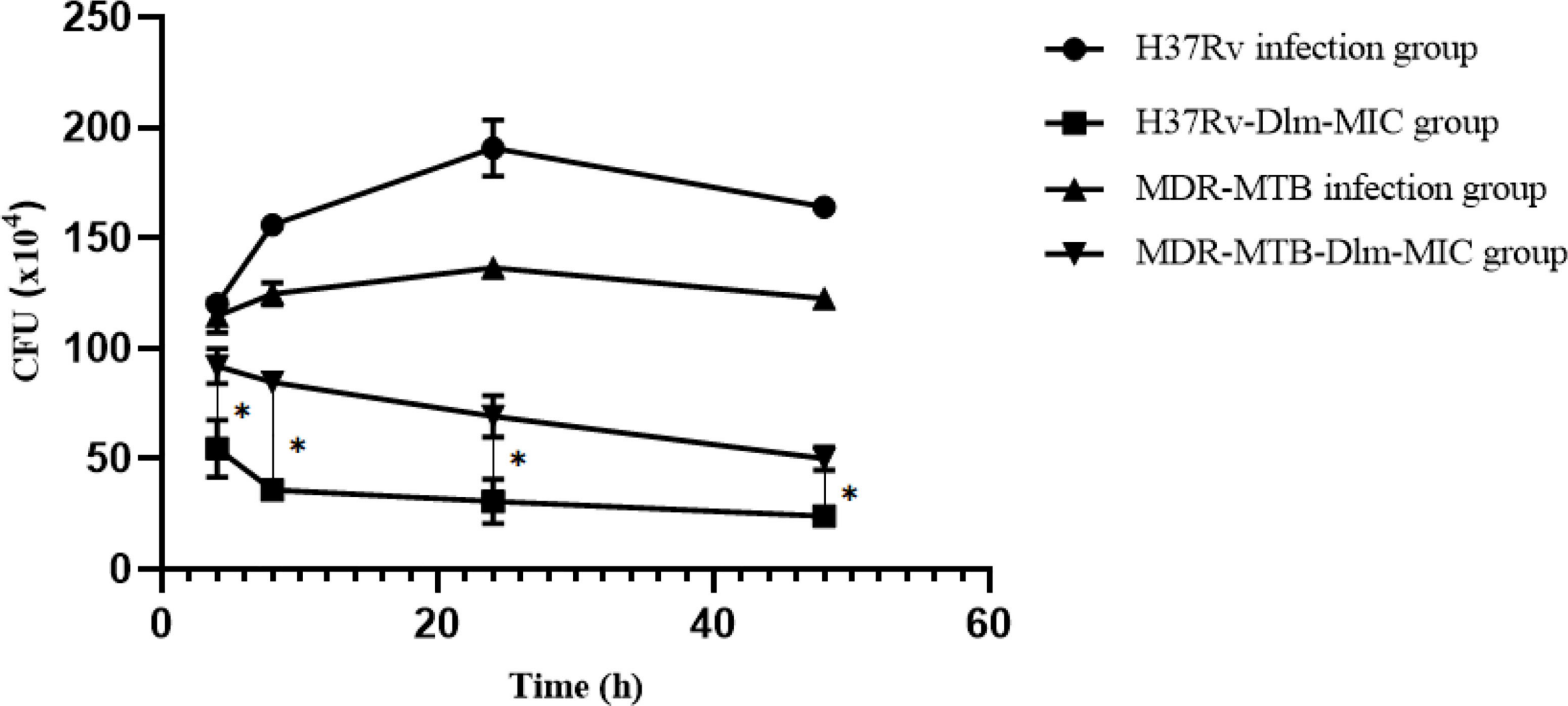
Intracellular CFU in the macrophages infected by MDR-MTB after Dlm was administered. *The P value of H37Rv-Dlm-MIC group and MDR-MTB-Dlm-MIC group was less than 0.05.

After Dlm was applied to macrophages infected by H37Rv or MDR-MTB strains with dose of MIC, the results of intracellular CFU were shown in **Figure 1**. As shown in **Figure 1**, the intracellular CFUs of MDR-MTB group at 4~48 h were higher than that of H37Rv group (*P<*0.05).

### The Level of Th1/Th2 Cytokines in the Culture Supernatant of Macrophages Infected by MDR-MTB Strains After Dlm Was Administered

After Dlm at MIC dose was applied to macrophages infected by H37Rv and MDR-MTB strains, the level of the culture supernatant of IL-12/23 p40 was shown in **Table 2**. As shown in **Table 2**, the level of IL-12/23 p40 of H37Rv and MDR-MTB group didn’t change before and after Dlm administration for 4~48 h, and the level of IL-12/23 p40 of MDR-MTB group at any time was similar to that of H37Rv group (*P*>0.05). In addition, the level of IL-12/23 p40 of H37Rv group at 48 h was lower than that of the infection group, while the level of IL-12/23 p40 of MDR-MTB group at 4 h was higher than that of the infection group (*P*<0.05).

**Table 2:** The level of IL-12/23 p40 in the culture supernatant of macrophages infected by MDR-MTB after Dlm was administered (pg/ml, $\bar{x}$ ± *s*).

	4 h before administration	4 h after administration	24 h after administration	48 h after administration	*F* test	*P* value
**H37Rv strains**						
Control	737.87 ± 93.61	722.80 ± 2.70	875.86 ± 21.11	867.81 ± 1.63	5.834	0.061
Dlm	737.87 ± 93.61	768.96 ± 30.59	831.21 ± 45.67	830.35 ± 10.34	1.451	0.354
* t* test	–	−2.126	1.255	5.060	–	–
* P* value	–	0.167	0.336	0.037	–	–
**MDR-MTB strains**						
Control	757.70 ± 52.47	722.54 ± 2.64	760.78 ± 0.61	752.17 ± 1.80	0.890	0.519
Dlm	757.70 ± 52.47	805.55 ± 25.35	731.81 ± 12.04	835.00 ± 91.68	1.445	0.355
* t* test	–	−4.606	3.398	−1.277	–	–
* P* value	–	0.044	0.077	0.330	–	–

After Dlm at MIC dose was applied to macrophages infected by H37Rv and MDR-MTB strains, the level of the culture supernatant of TNF-α was shown in **Table 3**. As shown in **Table 3**, the levels of TNF-α of H37Rv and MDR-MTB group at 24 and 48 h were higher than those of pre-administration group (*P*<0.05), while the level of TNF-α of H37Rv group at any time was similar and the level of TNF-α of MDR-MTB group at any time was similar to that of H37Rv group (*P*>0.05). In addition, the level of TNF-α of MDR-MTB group at any time was similar to that of the infection group (*P*>0.05).

**Table 3:** The level of TNF-α in the culture supernatant of macrophages infected by MDR-MTB after Dlm was administered (pg/ml, $\bar{x}$ ± *s*).

	4 h before administration	4 h after administration	24 h after administration	48 h after administration	*F* test	*P* value
**H37Rv strains**						
Control	307.95 ± 17.27	385.20 ± 1.52^#^	500.39 ± 0.37^#&^	436.28 ± 0.57^#&♣^	175.914	0.001
Dlm	307.95 ± 17.27	387.10 ± 53.63	434.00 ± 79.03	433.94 ± 6.50	2.989	0.159
* t* test	–	−0.050	1.188	0.506	–	–
* P* value	–	0.964	0.357	0.663	–	–
**MDR-MTB strains**						
Control	334.96 ± 9.33	402.47 ± 2.85^#^	451.83 ± 1.19^#&^	475.95 ± 0.54^#&♣^	320.338	0.001
Dlm	334.96 ± 9.33	396.89 ± 34.63	464.12 ± 10.24^#&^	454.01 ± 27.48^#^	13.223	0.015
* t* test	–	0.227	−1.686	1.129	–	–
* P* value	–	0.841	0.234	0.376	–	–

^#^The P value of 4h before administration and other group was less than 0.05. ^&^The P value of 4h after administration and other group was less than 0.05. ^♣^The P value of 24h after administration and other group was less than 0.05.

After Dlm at MIC dose was applied to macrophages infected by H37Rv and MDR-MTB strains, the level of the culture supernatant of IL-6 was shown in **Table 4**. As shown in **Table 4**, the levels of IL-6 of H37Rv and MDR-MTB group at 24 and 48 h were higher than those of pre-administration group (*P*<0.05), and the level of IL-6 of MDR-MTB group was similar to that of H37Rv group (*P*>0.05). In addition, the levels of IL-6 of MDR-MTB and H37Rv group at 24 and 48 h were lower than those of the infection group (*P*<0.05).

**Table 4:** The level of IL-6 in the culture supernatant of macrophages infected by MDR-MTB after Dlm was administered (pg/ml, $\bar{x}$ ± *s*).

	4 h before administration	4 h after administration	24 h after administration	48 h after administration	*F* test	*P* value
**H37Rv strains**						
Control	9.75 ± 2.91	11.05 ± 1.07	59.96 ± 0.35^#&^	87.06 ± 0.37^#&♣^	1171.658	0.001
Dlm	9.75 ± 2.91	6.68 ± 1.43	20.24 ± 0.00^#&^	28.85 ± 6.12^#&^	17.127	0.010
* t* test	–	3.457	158.880	13.419	–	–
* P* value	–	0.074	0.001	0.006	–	–
**MDR-MTB strains**						
Control	7.69 ± 0.00	13.45 ± 0.64^#^	77.87 ± 0.21^#&^	119.50 ± 0.84^#&♣^	19757.538	0.001
Dlm	7.69 ± 0.00	9.75 ± 2.91	22.38 ± 3.03^#&^	31.01 ± 3.08^#&♣^	35.643	0.002
* t* test	–	1.751	25.866	39.243	–	–
* P* value	–	0.222	0.001	0.001	–	–

^#^The P value of 4h before administration and other group was less than 0.05. ^&^The P value of 4h after administration and other group was less than 0.05. ^♣^The P value of 24h after administration and other group was less than 0.05.

After Dlm at MIC dose was applied to macrophages infected by H37Rv and MDR-MTB strains, the level of the culture supernatant of IL-10 was shown in **Table 5**. As shown in **Table 5**, the level of IL-10 of H37Rv and MDR-MTB group didn’t change before and after Dlm administration for 4~48 h (*P*>0.05), while the level of IL-10 of H37Rv group at 24 h was higher than that of pre-administration group and MDR-MTB group (*P*<0.05). In addition, the levels of IL-10 of MDR-MTB and H37Rv group at 4~48 h were lower than that of the infection group (*P*<0.05).

**Table 5:** The level of IL-10 in the culture supernatant of macrophages infected by MDR-MTB after Dlm was administered (pg/ml, $\bar{x}$ ± *s*).

	4 h before administration	4 h after administration	24 h after administration	48 h after administration	*F* test	*P* value
**H37Rv strains**						
Control	465.43 ± 5.95	528.99 ± 1.70^#^	582.81 ± 3.58^#&^	566.52 ± 0.25^#&♣^	425.322	0.001
Dlm	465.43 ± 5.95	493.75 ± 7.11	521.55 ± 19.25^#^	478.61 ± 8.33^♣^	8.837	0.031
* t* test	–	6.817	4.425	14.911	–	–
* P* value	–	0.021	0.047	0.004	–	–
**MDR-MTB strains**						
Control	525.71 ± 79.17	544.22 ± 0.80	571.63 ± 1.93	627.78 ± 4.40	2.517	0.197
Dlm	525.71 ± 79.17	462.52 ± 20.42	432.74 ± 7.41	505.53 ± 24.07	1.920	0.268
* t* test	–	5.654	25.645	7.065	–	–
* P* value	–	0.030	0.002	0.019	–	–

^#^The P value of 4h before administration and other group was less than 0.05. ^&^The P value of 4h after administration and other group was less than 0.05. ^♣^The P value of 24h after administration and other group was less than 0.05.

### Analysis of the Relationship Between the Level of Th1/Th2 Cytokines and Intracellular CFU in the Macrophages Infected by MDR-MTB Strains After Dlm Was Administered

**Figure 2:**
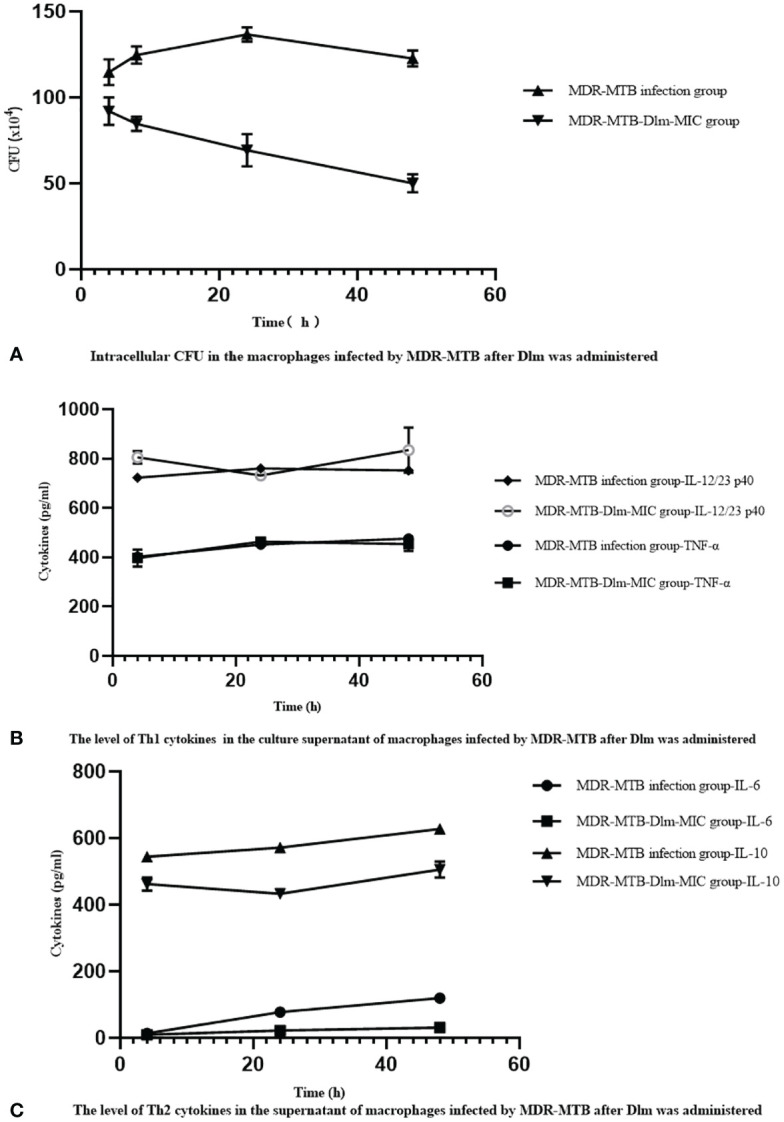
Dlm-mediated the changes of Th1/Th2 cytokines affects the bactericidal activity of the macrophages infected by MDR-MTB strains. **(A)** Intracellular CFU in the macrophages infected by MDR-MTB strains after Dlm was administered. **(B)** The level of Th1 (IL-12/23 p40 and TNF-α) cytokines in the culture supernatant of macrophages infected by MDR-MTB strains after Dlm was administered. **(C)** The level of Th2 (IL-6 and IL-10) cytokines in the culture supernatant of macrophages infected by MDR-MTB strains after Dlm was administered.

The relationship between the changes of Th1 cytokines IL-12/23 p40, TNF-α and, Th2 cytokines IL-6, IL-10 and the intracellular CFU in the macrophages infected by MDR-MTB strains after Dlm at MIC dose was administered was analyzed, and the analysis result was shown in **Figure 2**. As shown in **Figure 2**, the levels of IL-12/23 p40 and IL-10 were similar, and they didn’t change with the change of intracellular CFU at any time. The level of TNF-α at 24 and 48 h increased with the intracellular CFU decreasing. The level of IL-6 increased with the intracellular CFU decreasing, and reached the highest value at 48 h.

## Discussion

*Mycobacterium tuberculosis* (MTB) is a facultative parasite with macrophages as the host. Macrophages are derived from monocytes and have powerful phagocytic function. They are important immune effector cells against MTB invasion. It has been reported that MDR-MTB strains can lead to abnormal cellular immune function in the host so as to limit the killing and elimination of MDR-MTB strains (10, 11). There are few reports that Dlm influences the secretion of cytokines of the macrophages infected by MDR-MTB strains. Therefore, we observed whether cytokines are involved in the regulation of the bactericidal activity of Dlm.

As shown in **Table 1**, the intracellular CFU in the macrophages infected by MDR-MTB strains was lower than those of infection group (no-drug group) after Dlm at MIC at 4~48 h was administered (*P*<0.05), which suggested that Dlm had good bactericidal activity against MDR-MTB strains in macrophages. There are some bactericidal characteristics of Dlm against intracellular MDR-MTB strains in macrophages in this study. Firstly, Dlm had early bactericidal activity against intracellular MDR-MTB strains in macrophages because the intracellular CFU decreased by 20% at 4 h after Dlm was administered (**Table 1**). This result was consistent with the report by Andreas H. Diacon (12), which showed that the average sputum CFU was reduced by about 19% in 42 MDR-TB patients treated with Dlm for 14 days. It suggested that early use of Dlm can help to kill MDR-MTB and may reduce the resistance of bacteria. Secondly, the bactericidal activity of Dlm was time-dependent. The intracellular CFU in the macrophages infected by MDR-MTB strains decreased by 55% compared with before treatment at 48 h after administration with Dlm, which suggested that Dlm had delayed bactericidal effect. Andreas H. Diacon (12) also found that the CFU of smear-positive pulmonary tuberculosis patients decreased rapidly after treatment with Dlm for 2 days. It was suggested that Dlm may accumulate in the cell and has potential activity against the intra- and extracellular bacilli (13). Thirdly, the bactericidal activity of Dlm was concentration-dependent. As shown in **Table 1**, increasing the dose of Dlm to 10MIC and 20MIC at 48 h could further reduce the intracellular CFU, and the intracellular CFU at 20MIC at any time was lower than that at 10MIC (*P*<0.05). It was consistent with the report of Andreas H. Diacon (12), which showed that sputum CFU treated with Dlm at 300 mg/day for 14 days was lower than that treated with 100 and 200 mg/day. It suggested that reasonable therapeutic dose can help to ensure the bactericidal activity of Dlm. Fourth, the bactericidal activity of Dlm against MDR-MTB strains was weaker than that of H37Rv group. As shown in **Figure 1**, the intracellular CFU in the macrophages infected by MDR-MTB strains was higher than that in the macrophages infected by H37Rv strains at 4~48 h after administration with Dlm. To analyze the reasons, one was that MDR-MTB strains may escape the clearance of macrophages through relevant mechanisms. The other reason was that the phenotype and molecular drug sensitivity tests of Dlm were not performed for MDR-MTB clinical isolates in this study, so that the influence of primary drug resistance on the results could not be excluded.

Unlike most pathogenic bacteria, the main host cells attacked by MTB are the macrophages in the lungs. After MTB infects macrophages, the macrophages mainly inhabit or kill intracellular MTB by secreting cytokines, changing metabolic pathways, autophagy, pyrolysis, and other mechanisms (14). In this study, four Th1/Th2 cytokines were selected to explore the expression and effects caused by Dlm on the phagocytosis infected by MDR-MTB strains.

In terms of Th1 cytokines, IL-12/23 p40 and TNF-α have pro-inflammatory effect, which can promote the production of IFN-γ or coordinate with IFN-γ to directly or indirectly promote the activation of macrophages so as to play a role in phagocytizing, inhibiting, and killing MTB (15, 16). **Tables 2**, **3** showed that the level of IL-12/23 p40 in the culture supernatant of macrophages infected by MDR-MTB strains was higher than that of infection group at 4 h after administration with Dlm, while the level of TNF-α at any time was similar to that of the infection group. It suggested that Dlm, just as a immunopotentiator, may promote the production of IL-12/23 p40 to coordinate and promote the bactericidal effect of macrophages. However, this promotion effect of Dlm lasted for a short time. There may be some reasons for that. One is that it may be related to treatment with one-time so that the stimulating effect on the secretion of cytokines was weakened after the drug concentration decreased. Second, there may be other mechanisms involved in the delayed bactericidal effect of Dlm, which needs to be further discussed. In addition, the levels of IL-12/23 p40 and TNF-α of MDR-MTB group at any time were similar to that of H37Rv group (**Table 2**, **3**), suggesting that the immunomodulatory effect of Dlm may only target MTB and macrophages and has little to do with the drug resistance of MTB.

In terms of Th2 cytokines, IL-6 and IL-10 can inhibit the fusion of phagocytic lysosomes and autophagy induced by IFN-γ in macrophages so as to provide favorable conditions for the survival of MTB *in vivo* (17, 18). **Tables 4**, **5** showed that the level of IL-6 at 24~48 h and IL-10 at 4~48 h of MDR-MTB group were respectively lower than those of infection group after administration with Dlm, suggesting that Dlm might have immunosuppressive effect on IL-6 and IL-10, which could inhibit their secretion and expression so as to promote the formation of the immune response dominated by Th1 cell indirectly and the elimination of MDR-MTB. However, it couldn’t be ruled out the influence of which the decrease of bacterial load could reduce the stimulation to the production of Th2 cytokines after Dlm killed MDR-MTB in the macrophages. In addition, the level of IL-10 of MDR-MTB group was lower than that of H37Rv group at 24 h after administration with Dlm, while the level of IL-10 of MDR-MTB group at any time was similar to that of H37Rv group. It was suggested that the effect of Dlm on different cytokines was not consistent in the macrophages infected by MDR-MTB and H37Rv strains, and the mechanism remains to be further explored, and the influence of the level of inconsistent cytokines in the two cell models after infection cannot be completely excluded.

We observed the influence of four cytokines on the phagocytosis and bactericidal activity of macrophages infected by MDR-MTB strains in **Figure 2**. The levels of IL-12/23 p40 were similar at any time and didn’t change with the change of intracellular CFU. The level of TNF-α increased at 24 and 48 h, accompanied by the decrease of intracellular CFU, while the level of TNF-α was similar to that of the infection group at any time. It suggested that this change was related to the cellular immune response of macrophages infected by MDR-MTB strains and had nothing to do with the effect of Dlm. As for Th2 cytokines, the level of IL-6 increased with decrease of intracellular CFU, but the increasing level was significantly lower than that of the infection group (*P*<0.01); while the level of IL-10 did not change with the change of intracellular CFU, and its expression level was also significantly lower than that of the infection group (*P*<0.01). It showed that Dlm might inhibit the secretion and expression of IL-6 and IL-10. It may be related to Dlm downregulating the expression of IL-6 and IL-10, weakening the negative impact of Th2 immune response on the phagocytosis and bactericidal activities of macrophages, thereby contributing to the reduction of the bacterial load in macrophages. It is suggested that Dlm might not only inhibit the synthesis of MTB cell wall as a bactericide but also inhibit Th2 immune response as an immunomodulator. Dlm as immunomodulatory that triggers multiple immune pathways may strengthen MDR-TB treatment and has the prospective capability to change how we treat MDR-TB (19, 20).

There were limitations in our study. The results could only illustrate the effect of Dlm on the macrophages infected only by similar MDR-MTB clinical isolates. We should expand the sample size and drug resistance types to further study in order to obtain more reliable results.

In conclusion, Dlm had strong bactericidal activity against intracellular MDR-MTB of macrophages infected by MDR-MTB. However, its bactericidal activity was weaker than that against drug-susceptible tuberculosis strains. There were time-dependent and concentration-dependent for its bactericidal activity. In addition, Dlm might have immunomodulatory effect, inducing low expression of Th2 cytokines IL-6 and IL-10 at different times after administration.

## Data Availability Statement

The datasets used and/or analyzed during the current study are available from the corresponding author on reasonable request.

## Ethics Statement

This research was approved by the Ethics Committee of Beijing Chest Hospital, Capital Medical University with the ethical code number YJS-2021-009.

## Author Contributions

X-LL: study design, data acquisition, analysis of results, and manuscript preparation. L-PP, Z-DZ, and QL: study design, analysis of results, critical review, and modification of the manuscript. T-TL, J-TG, H-YJ, C-ZZ, Z-HL, JD, QS, and WS: statistical analysis and critical review of the manuscript. All authors contributed to the article and approved the submitted version.

## Funding

This study was financially supported by Research and Evaluation of New Protocols and Technologies for the Treatment of Drug-resistant Tuberculosis (2018ZX10722301-001) and the Beijing Municipal Science and Technology Major Project (D181100000418005).

## Conflict of Interest

The authors declare that the research was conducted in the absence of any commercial or financial relationships that could be construed as a potential conflict of interest.

## Publisher’s Note

All claims expressed in this article are solely those of the authors and do not necessarily represent those of their affiliated organizations, or those of the publisher, the editors and the reviewers. Any product that may be evaluated in this article, or claim that may be made by its manufacturer, is not guaranteed or endorsed by the publisher.

## Abbreviations

MTB, *Mycobacterium tuberculosis*; MDR-MTB, multidrug-resistant *Mycobacterium tuberculosis*; MDR-TB, multidrug-resistant tuberculosis; RR-TB, rifampicin-resistant tuberculosis; Dlm, Delamanid; CFU, colony-forming unit; MIC, minimal inhibitory concentration.
